# Vancomycin-Resistant Enterococci: Current Understandings of Resistance in Relation to Transmission and Preventive Strategies

**DOI:** 10.3390/pathogens13110966

**Published:** 2024-11-05

**Authors:** Ivana Mareković, Manda Markanović, Joško Lešin, Mario Ćorić

**Affiliations:** 1 Clinical Department of Clinical Microbiology, Infection Prevention and Control, University Hospital Centre Zagreb, 10000 Zagreb, Croatia; 2 School of Medicine, University of Zagreb, 10000 Zagreb, Croatia; 3 Department of Obstetrics and Gynaecology, University Hospital Centre Zagreb, 10000 Zagreb, Croatia

**Keywords:** vancomycin, enterococci, antimicrobial resistance, virulence genes, contact isolation

## Abstract

Due to the limited treatment options and increased mortality rates, infection prevention and control strategies have been implemented for many years to mitigate dissemination of vancomycin-resistant enterococci (VRE) within healthcare settings. The overview provides an insight into the most recent research, particularly the pathogen’s resilience in the healthcare environment, and the critical need for infection control strategies, which are currently being scrutinized by some researchers. The notable resilience of enterococci to various environmental conditions highlights the necessity for investigations into innovative technologies capable of effectively targeting the biofilm produced by enterococci on hospital surfaces. A critical approach to traditional infection control strategies is becoming more accepted worldwide, taking into account the epidemiological situation in the given healthcare setting as well as specific characteristics of a patient. For certain high-risk patient populations, traditional infection control strategies including CP and screening should not be omitted. Additionally, further investigation into the resistance mechanisms of available antimicrobial agents is essential, as is research into their potential association with specific successful clones through WGS genotyping, to pre-emptively mitigate their spread before it escalates.

## 1. Introduction

*Enterococcus* species constitute an essential and normal part of the intestinal microbiota in both healthy humans and animals. Over 60 different species of *Enterococcus* have been recognized, with *E. faecalis* and *E. faecium* being the most prevalent in the human gastrointestinal tract. While enterococci play a beneficial role in the microbiome, they can also act as opportunistic pathogens, leading to severe and potentially fatal infections, including urinary tract infections, surgical site infections, bloodstream infections, and endocarditis [1]. The predominant cause of enterococcal infections is *E. faecalis*; however, the increasing prevalence of multidrug-resistant (MDR) *E. faecium* has contributed to a rise in nosocomial infections, particularly bloodstream infections associated with this species [2]. Throughout much of the 20th century, perceptions of enterococci as significant pathogens were often unclear, with some researchers suggesting that their pathogenic potential was limited. This perspective was influenced by the fact that infections from which enterococci were isolated were often polymicrobial, that these bacteria are commonly found in the gastrointestinal tracts of humans, and that certain enterococcal strains have been marketed as probiotics [3,4]. The first clinical isolates of vancomycin-resistant enterococci (VRE) were reported in 1986 by Leclercq et al. in France and Uttley et al. in the UK. Both *E. faecalis* and *E. faecium* were involved in these outbreaks, exhibiting significant resistance to teicoplanin and vancomycin [5].

Enterococci are currently acknowledged as pathogenic bacteria that pose significant treatment challenges with conventional antimicrobial therapies [6]. Additionally, they are classified among the primary pathogens responsible for bacterial nosocomial infections, collectively referred to as “ESKAPE,” which encompasses *E. faecalis*, *Staphylococcus aureus*, *Klebsiella pneumoniae*, *Acinetobacter baumannii*, *Pseudomonas aeruginosa*, and *Enterobacter* spp. [7]. Vancomycin is a fundamental antimicrobial agent used for treatment of severe infections caused by Gram-positive bacteria including septicemia, skin and soft tissue infections, bone infections, lower respiratory tract infections, and endocarditis, as well as those caused by methicillin-resistant *Staphylococcus aureus* (MRSA). Its oral administration has a role in the treatment of *Clostridioides difficile*-associated diarrhea [8]. Infections caused by vancomycin-resistant *E. faecium* are associated with increased mortality rates compared to those caused by vancomycin-susceptible strains. Specifically, the 30-day and 90-day mortality rates for patients infected with vancomycin-resistant *E. faecium* were 57.7% and 69.2%, respectively, in contrast to 38.7% and 47.1%, respectively, for their susceptible counterparts. Furthermore, patients with vancomycin-resistant *E. faecium* infections experienced a significantly prolonged duration of hospitalization [9].

Treatment alternatives for infections caused by vancomycin-resistant enterococci (VRE) are constrained, with linezolid and daptomycin representing the most commonly utilized antimicrobial therapies. Nonetheless, resistance to these agents, albeit at a low frequency, is beginning to surface, prompting the exploration of additional treatment options [10]. Due to the restricted availability of treatment alternatives and elevated mortality rates, infection prevention and control strategies have been implemented for many years to mitigate the dissemination of these pathogens within healthcare settings. Recently, however, the effectiveness and rationale behind these approaches—such as contact precautions and active screening—are being questioned and, in certain hospitals, particularly in the United States and Australia, are even being discontinued [11,12].

The objective of this overview is to provide an understanding of the most recent research and methodologies concerning VRE, particularly in relation to the implications and importance of this resistance within hospital settings. It presents recent foundational knowledge concerning resistance genes, their worldwide spread, virulence factors, genotyping, and the risks of resistance to existing treatment modalities. Emphasis is placed on the pathways of transmission, the pathogen’s resilience in the healthcare environment, and the critical need for infection control strategies, especially contact precautions and active screening, which are currently being scrutinized by some researchers.

## 2. Global Distribution of Vancomycin-Resistant Enterococci

The World Health Organization, the Centers for Disease Control and Prevention, and the European Center for Disease Prevention and Control have identified vancomycin-resistant enterococci (VRE) as a global health threat [13,14,15]. A significant rise in the prevalence of vancomycin resistance, particularly in *E. faecium*, has been documented across various countries [16]. In Europe, this issue is especially severe in the central, southern, and eastern regions, where recent surveillance indicates that vancomycin resistance affects between 10% and 50% of *E. faecium* isolates. The most recent ECDC annual epidemiological report from 2022 reveals that the population-weighted mean percentage of vancomycin-resistant *E. faecium* isolates in the EU/EEA increased from 16.2% in 2018 to 17.6% in 2022 [15]. However, there is considerable variability among countries. Research conducted in Germany indicates a rising prevalence of VRE in hospitals, exceeding 20%, while studies in the Netherlands report resistance rates below 1%, likely attributable to differences in antibiotic usage and adherence to infection prevention and control protocols [17]. Additionally, several other European nations are experiencing elevated rates of vancomycin resistance among *E. faecium* isolates, with Lithuania (66.4%), Cyprus (51.2%), Romania (44.5%), Greece (41.1%), Hungary (40.7%), Croatia (39.8%), Slovakia (34.7%), and Ireland (27.6%) reporting significant figures in 2021. Around 30% of healthcare-associated enterococcal infections in the United States exhibit resistance to vancomycin, as reported by the CDC. In Australia, the prevalence of vancomycin resistance in *E. faecium* escalated to one of the highest globally, reaching 39.3% to 46.8% of clinical isolates in 2017 [18].

The *vanA* and *vanB* operons are the most prevalent and clinically significant among VRE strains. Currently, the *vanA* genotype is recognized as the most widespread globally; however, this pattern is undergoing transformation. Recent studies in hospitals in the Netherlands and Germany indicate a notable shift in the molecular epidemiology of vancomycin-resistant *E. faecium*, moving from a predominance of the *vanA* genotype to an increasing prevalence of the *vanB* genotype over time [17]. In Germany, the last decade has seen the *vanB* genotype surpass *vanA* as the dominant strain [19]. A similar transition from *vanA* to *vanB* clusters has been documented in Denmark, based on isolates collected between 2019 and 2022 [20]. Furthermore, a high prevalence of the *vanA* gene among VRE has been reported in several Asian nations, particularly in Republic of Korea, Japan, and China [21]. In a health perspective including human, animal and environmental samples, the overall prevalence of VRE in Africa was 26.8% with the highest prevalence in South Africa (76.8%) [22]. Genotype analysis showed low detection of *vanB* and the absence of the *vanA* genotype in Southwest Nigeria [23].

## 3. Mechanisms and Transfer of Antimicrobial Resistance in Enterococci

Glycopeptide antimicrobial agents represent a class of compounds that target Gram-positive bacteria by disrupting their cell wall synthesis. This disruption occurs through the agents’ binding to the D-alanine-D-alanine (D-ala-D-ala) site, which inhibits the cross-linking of the peptidoglycan layer in the bacterial cell wall. The structural features of glycopeptides limit their efficacy against Gram-negative bacteria, as these characteristics obstruct their ability to traverse the outer membrane of this bacterial group. Vancomycin, the first glycopeptide to be identified, was derived from the soil fungus *Streptomyces orientalis* in Borneo in 1957 by organic chemist E.C. Kornfield. The compound received approval from the Food and Drug Administration (FDA) in 1958 and was named “vancomycin”, a term derived from the word “vanquish” [24,25]. Approximately three decades later, a related natural product known as teicoplanin was isolated from *Actinoplanes teichomyceticus*, featuring a hydrophobic substituent termed lipoglycopeptide. Subsequently, new semisynthetic lipoglycopeptides were developed, including dalbavancin, oritavancin, and telavancin [25].

Glycopeptide resistance in enterococci was first identified nearly four decades ago. This resistance emerged due to various factors, including agricultural practices and the incorporation of avoparcin into animal feed in Europe and other regions. The mechanism of glycopeptide resistance is characterized by the production of enzymes that modify the D-ala-D-ala binding site to either D-alanine-D-lactate (D-ala-D-lac) or D-alanine-D-serine (D-ala-D-ser), which leads to a significant decrease in glycopeptide binding affinity, approximately 1000-fold and 7-fold, respectively [25,26].

**Figure 1:**
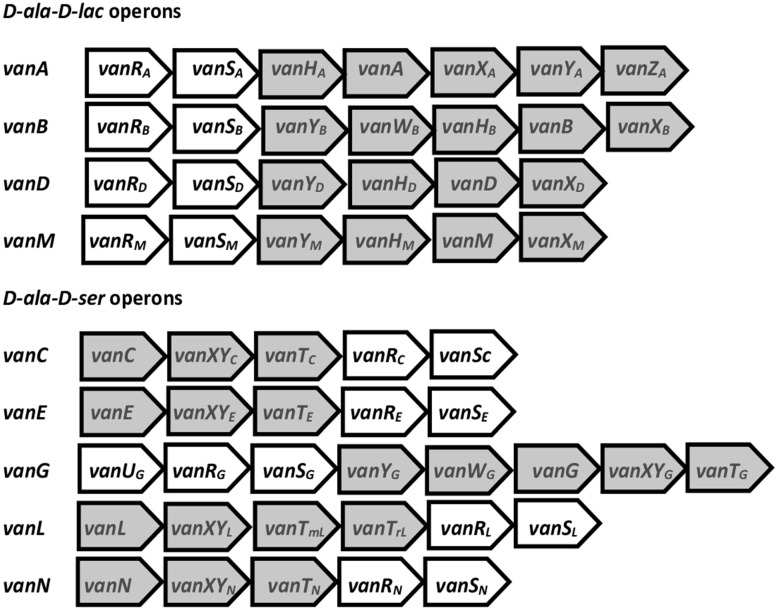
The organization of the nine *van* operons in enterococci. The *D*-*ala*-*D*-*lac* operons (*vanA*, *vanB*, *vanD*, and *vanM*) and *D*-*ala*-*D*-*ser* operons (*vanC*, *vanE*, *vanG*, *vanL*, and *vanN*) are shown. These operons consist of regulatory genes (depicted in white) and structural genes (depicted in gray) that encode proteins crucial for glycopeptide resistance. The genes and their corresponding proteins are as follows: *vanR*, a response regulator; *vanS*, a sensor histidine kinase; *vanA*, *vanB*, *vanD*, *vanM*, *vanC*, *vanE*, *vanG*, *vanL*, and *vanN*, which are ligases that synthesize D-ala dipeptides with lactate or serine; *vanH*, a dehydrogenase; *vanX*, a dipeptidase; *vanY*, a carboxypeptidase; *vanZ*, whose function remains unknown, but is implicated in teicoplanin resistance; *vanW*, also of unknown function; *vanXY*, which acts as a dipeptidase/carboxypeptidase; *vanT*, a serine racemase; *vanT_m_*, a membrane domain of serine racemase; and *vanT_r_*, the racemase domain of serine racemase [26,27,28].

Resistance genes to glycopeptides are organized within *van* operons located on mobile genetic elements (MGEs). These operons include regulatory genes that control the activation of ligase genes, which are crucial for glycopeptide resistance, with *vanA* and *vanB* being the most common and responsible for the majority of VRE outbreaks in humans. Currently, the following nine distinct *van* operons have been identified: *vanA*, *vanB*, *vanC*, *vanD*, *vanE*, *vanG*, *vanL*, *vanM*, and *vanN*, each varying in their levels of glycopeptide resistance. A schematic diagram of these *van* operons is provided in Figure 1. The regulatory genes include *vanR* (response regulator), *vanS* (sensor histidine kinase), and *vanU* (transcriptional activator). The *vanS* acts as a membrane-bound sensor that is activated upon the presence of glycopeptides, subsequently transferring a phosphoryl group to *vanR*, which initiates the co-transcription of the *vanH*, *vanA*, *vanX*, and *vanY* genes [27]. In *vanA*, the operon structural genes specifically encode the production of enzymes involved in resistance development—dehydrogenase that converts pyruvate to lactate (*vanH*), ligase that forms D-ala-D-lac dipeptide (*vanA*), dipeptidase that cleaves D-ala-D-ala (*vanX*), and D-carboxypeptidase hydrolyzes the terminal D-ala of pentapeptide precursors that are produced if elimination of the D-ala-D-ala is not complete (*vanY*). The *vanZ* gene is a gene related to teicoplanin resistance. The genetic organization of the *vanB* operon is similar except it lacks a homolog of *vanZ* and instead contains *vanW* with a still unresolved role in the resistance development [28].

The phenotypic effects resulting from the transcription of operons vary based on the specific type of operon and the previously discussed reductions in the binding affinity of D-ala-D-lac and D-ala-D-ser, which modify the binding sites. The *vanA* operon is associated with a high degree of resistance to both vancomycin and teicoplanin. In contrast, the *vanB* resistance mechanism activates regulatory proteins solely in response to vancomycin, not teicoplanin, leading to strains exhibiting high-level resistance exclusively to vancomycin. The *vanC* resistance type, observed in *E. gallinarum*, *E*, *casseliflavus*, and *E. flavescens*, provides low-level resistance to vancomycin but does not confer resistance to other glycopeptides. Likewise, strains classified as *vanE* and *vanG*, which also exhibit the D-ala-D-ser change of a binding site, demonstrate only intermediate-level resistance to vancomycin. The *vanM* is genetically and phenotypically similar to *vanA*, *vanB*, and *vanD*, whereas both *vanL* and *vanN* are similar to *vanC* (Table 1) [27,29].

A recently identified subtype of VRE, referred to as vancomycin-variable enterococci (VVE), has been documented. The prevalence of these organisms remains uncertain, as they elude detection through conventional phenotypic methods. Notably, VVE strains are susceptible to vancomycin (designated as VVE-S) and possess the *vanA* genotype. However, it is noteworthy that upon treatment with vancomycin or teicoplanin, these strains can acquire vancomycin resistance (designated as VVE-R), which may lead to therapeutic failure [32].

The *E. faecalis* and *E. faecium* can easily acquire new resistance and virulence genes located on different MGEs including plasmids, transposons, and bacteriophages by horizontal gene transfer (HGT). These elements enable enterococci to adapt to hospital environment and turn into multidrug-resistant microorganisms. For example, phages in *E. faecalis* are able to transfer resistance genes and the *gelE* virulence gene via transduction [33].

The One Health approach considers antimicrobial resistance as a problem affecting humans, animals, and the environment. According to WHO, 75% of human infections have their source in animals as a consequence of intense urbanization, trade, travel, and agriculture. Enterococci are no exception, and because they are ubiquitous in humans, animals, and the environment, can easily acquire and disseminate resistance genes, and even are considered to be an ideal One Health indicator of antimicrobial resistance [34]. In the study by Zaheer et al., out of a total of 8430 isolates, *E. faecalis* and *E. faecium* were the predominant enterococcal species in clinical human isolates and urban wastewater (99% and 90%, respectively), and *E. hirae* are predominant in cattle (92%) [35]. Different types of food—mostly cheese and poultry meat—can be contaminated by enterococci as a consequence of poor hygiene. Additionally, enterococcal thermostable amines resistant to conditions used in food technology (temperature, pH, salinity), can cause allergic reactions and poisoning. Similar resistance profiles have been described in clinical and food isolates meaning that food chain between environment and humans is a one possible transmission route for antimicrobial resistance further potentiated by intensive trade [36]. Furthermore, the most prevalent genetic lineages, specifically ST17 (CC17), which are linked to human infections, were identified in products derived from swine and poultry. The transmission of particular genetic lineages between animals and humans has also been documented [37].

Enterococci also exhibit intrinsic resistance to various classes of antimicrobial agents. Their intrinsic resistance to beta-lactams arises from the presence of low-affinity penicillin-binding proteins (PBPs), resulting in low-level penicillin resistance and moderate to high-level cephalosporin resistance. Additionally, their low-level resistance to aminoglycosides is due to the inability of these agents to penetrate the bacterial cell as well as due to some of the aminoglycoside-modifying enzymes (low-level tobramycin and kanamycin resistance due to Aac(6′)-Ii and ribosome-modifying methyltransferase). Nevertheless, when aminoglycosides are administered in combination with cell wall-active agents, a bactericidal synergistic effect is observed. Furthermore, enterococci are naturally resistant to clindamycin, quinupristin, and dalfopristin (mainly due to the ABC-efflux pump). The combination of sulfamethoxazole–trimethoprim is also ineffective against enterococcal infections, as these bacteria can assimilate folic acid from their surroundings, thereby circumventing the inhibitory action of sulfamethoxazole–trimethoprim [38].

## 4. Virulence Genes in Vancomycin-Resistant Enterococci

Enterococci are typically regarded as less pathogenic compared to *S. aureus* or Gram-negative bacteria. Consequently, invasive infections caused by VRE predominantly arise in individuals with significantly compromised health due to pre-existing medical conditions. The ultimate prognosis for these patients is frequently influenced by their underlying health issues, with VRE infection potentially representing a terminal event in those with a markedly unfavorable outlook [39].

Numerous virulence genes associated with enterococci that may contribute to disease development in humans have been identified (Table 2). The most important enterococcal virulence determinants are the aggregation substance (*asa1*), gelatinase (*gelE*), cytolysin (*cylA*), enterococcal surface protein (*esp*), hyaluronidase (*hyl*), and collagen-binding protein (*acm*). Their roles have been elucidated through studies conducted on both humans and animals [40,41]. A detailed overview of various virulence determinants, their encoding genes, mechanisms of action, and their relevance in the pathogenesis of enterococcal infections is presented in Table 1. Notably, enterococcal surface protein and hyaluronidase are virulence factors that are specific to *E. faecium*. Strains of vancomycin-resistant *E. faecium* that have emerged in hospitals across the USA, Europe, and Australia frequently carry specific virulence genes, such as *esp* and *hyl*, which are absent in non-epidemic or animal-derived isolates, as well as in food or sewage samples [27]. These genes are believed to reside within a putative pathogenicity island and are regarded as indicators of nosocomial epidemics, facilitating the acquisition of antimicrobial resistance genes and the proliferation of vancomycin-resistant *E. faecium* in healthcare settings. It is well established that *Enterococcus* species possess highly effective gene transfer mechanisms, and virulent genes are often linked to highly transmissible plasmids, which may have facilitated the spread of virulent genes into less virulent strains of *E. faecium* [42].

In addition to the immediate cytotoxic effects caused by cytolysin, the majority of the elements associated with enterococcal virulence in animal models are related to the colonization of tissues, including factors such as enterococcal surface protein and collagen-binding protein. The *Enterococcus* species exhibit a notable ability to form biofilms, which contributes to their survival in various environments and their role in the establishment of chronic bacterial infections. Factors such as gelatinase, aggregation substance, and enterococcal surface protein are linked to biofilm development and have been identified in both commensal and clinical isolates of *Enterococcus* species, with differing prevalence rates observed [45].

Research on the distribution of virulence genes in enterococci is somewhat limited, particularly studies that examine the differences in virulence gene presence between vancomycin-susceptible enterococci (VSE) and VRE, as well as those that investigate the virulence gene profiles of colonizing versus invasive clinical isolates. In the investigation conducted by Biswas et al., it was found that the *gelE*, *esp*, and *hyl* genes were present in significantly greater quantities in VRE clinical isolates compared to their VSE counterparts. Additionally, in fecal isolates, the *gelE*, *esp*, and *asa1* genes were also found to be significantly more prevalent [30]. In a study conducted by Cho et al., it was observed that the *hyl* genes were more prevalent in vancomycin-resistant *E*. *faecium* [46]. This prevalence may be attributed to the presence of both the *vanA* and *hyl* genes on the same plasmid. Conversely, a more recent investigation found that the *hyl* gene was absent in VRE [47]. Doss Susai’s research revealed that among vancomycin-resistant *E. faecalis*, the *gelE* gene was the most common at 76%, followed by *asaI* at 73%, *esp* at 70%, *ace* at 68%, *cylA* at 62%, and *hyl* at 5%. In the case of vancomycin-resistant *E. faecium*, *gelE* was also predominant at 69%, succeeded by *esp* at 63%, *asaI* at 59%, and both *cylA* and *ace* at 56%, with *sprE* at 48% and *hyl* at 30% [48]. Furthermore, virulence genes such as *asa1*, *cylA*, *gelE*, *esp*, and *hyl* were found to be more frequently associated with clinical VRE compared to fecal VRE, with the exception of *esp*, which was more prevalent in fecal samples. Notably, the *hyl* gene was significantly more common in clinical VRE than in fecal VRE (*p* = 0.043). It is important to highlight that this study encompassed not only *E. faecalis* and *E. faecium* but also other enterococcal species, including *E. gallinarum*, *E. raffinosus*, *E. malodoratus*, *E. solitaries*, and *E. durans* [41].

## 5. Genotyping Methods and the Importance of Specific Genetic Profiles

The limited therapeutic options available for VRE infections necessitate a comprehensive understanding of VRE transmission networks within hospital settings. This knowledge is crucial for effectively managing and mitigating the emergence and spread of these pathogens. The identification of clonal transmission of VRE within healthcare facilities highlights the critical need for robust infection control strategies and antimicrobial stewardship initiatives to prevent the dissemination of these highly resistant organisms among patients and across different hospital rooms.

The inquiry into the clinical significance of specific *E. faecium* isolates, as opposed to those that merely function as commensal organisms, has posed a considerable challenge for both clinicians and researchers. Genotyping methodologies have revealed the existence of two distinct evolutionary populations *of E. faecium*, designated as clade A and clade B. Clade A is predominantly composed of isolates associated with hospital environments and animal sources, whereas clade B encompasses isolates that are primarily linked to community settings. These two clades were first identified approximately twenty years ago through the use of amplified fragment length polymorphism (AFLP) and have since been corroborated by multi-locus sequence typing (MLST) and whole genome sequencing (WGS), demonstrating notable differences in their characteristics [21,49,50].

Clade A is subdivided into the following two distinct groups: clade A1, which is composed of clinical isolates, and clade A2, which includes commensal isolates from both animals and humans that infrequently result in human infections. Each of these clades possesses genetic factors associated with virulence and resistance [51]. Researchers utilized mutation rates to approximate the timing of the divergence among the A1, A2, and B clades. These analyses suggest that the separation between clades A and B occurred approximately 2776 ± 818 years ago, while the divergence between clades A1 and A2 took place around 74 ± 30 years ago. This evidence implies that clade A, predominantly composed of animal isolates, diverged from the human commensal clade B roughly 3000 years ago, likely driven by factors such as increasing urbanization, improvements in sanitation, and the domestication of animals. Furthermore, the advent of antimicrobial usage 75 years ago is believed to have contributed to the divergence of the hospital lineage (clade A1) from the primarily animal isolate clade A. Notably, clade A1 strains have also been identified in pet dogs, supporting earlier research that indicates a connection between hospital strains and domestic animals [38]. Furthermore, VRE has been identified in various companion animals, including dogs, cats, horses, parrots, pheasants, and rabbits [52]. Clade B predominantly consists of isolates that are commensal to humans. Given their genetic resemblance to *E. lactis*, there is a proposal to reclassify these isolates within this newly defined species [51].

Through the use of MLST characterizing the loci within seven *E. faecium* housekeeping genes (*atpA*, *ddl*, *gdh*, *purK*, *gyd*, *pstS*, *and adk*), *E. faecium* can be categorized into various sequence types (STs) [53]. Among them, ST17 is being recognized as the original clone of hospital-associated (HA) isolates, forming clonal complex 17 (CC17) associated with hospital ward outbreaks around the globe [49,54].

Pulsed field gel electrophoresis (PFGE) has long been regarded as the gold standard for bacterial typing; however, whole genome sequencing (WGS) has emerged as a rapid and cost-effective alternative. Many researchers now view WGS as the new gold standard for VRE typing and it is progressively supplanting PFGE. In the research conducted by Pinholt et al., PFGE corroborated the groupings and subgroupings established through single nucleotide polymorphism (SNP) analysis. Nevertheless, it also misclassified certain isolates and failed to differentiate some WGS subgroups, leading to a lack of precision in identifying transmission routes compared to WGS [55].

The WGS analysis conducted by Pinholt et al. elucidated the transmission dynamics of VRE during several hospital outbreaks in the Capital Region of Denmark between 2012 and 2015. The predominant clonal group identified was ST80, which constituted 40% of the isolates and was linked to the initial local hospital outbreak. Additionally, the *vanA* plasmid, designated pV24-5, was detected in 81% of the VRE isolates. The researchers noted the presence of both susceptible and resistant *E. faecium* isolates across various clonal groups, reinforcing the notion that, alongside clonal expansion, the ST80 group effectively propagated the pV24-5 plasmid through horizontal gene transfer to pre-existing hospital-adapted susceptible *E. faecium*, leading to the emergence of new VRE clones [56]. The likely reason lies in the *E. faecium* genome’s notable recombination rate, with studies indicating that as much as 44% of its core genome is influenced by recombination, which encompasses five out of the seven MLST alleles. This phenomenon could result in inaccuracies during phylogenetic analyses, shedding light on the significant number of potential misclassifications observed with PFGE in contrast to WGS and MLST [57].

Moreover, MLST faces certain challenges, including its limited ability to distinguish between strains. Some VRE isolates have been discovered that do not possess the *pstS* gene, which is one of the seven loci used in MLST. Additionally, recombination has been demonstrated to influence the VRE genome in critical areas of these loci. The ever-changing landscape of the *E. faecium* genome, along with the ongoing exchange of accessory genes and horizontal gene transfer, highlights the inadequacies of MLST as a typing technique [58]. The consistent and immediate application of WGS to uncover new or previously unnoticed transmission networks and potential reservoirs of dominant pathogens in a hospital setting has emerged as a reliable strategy for infection control monitoring. This approach can significantly help in curbing the spread of pathogens, particularly those that are multidrug-resistant, within the healthcare environment.

## 6. Treatment Options, Risk of Resistance Development, and New Potential Therapies

The present antimicrobial therapy for infections caused by VRE predominantly utilizes the following three antimicrobial agents: linezolid, daptomycin, and tigecycline.

Linezolid, a bacteriostatic agent belonging to the oxazolidinone class, received its approval for clinical use in 2000 and has since become a cornerstone in the treatment of infections caused by VRE. This antibiotic functions by binding to the V domain of the 23S rRNA within the 50S ribosomal subunit, effectively blocking the formation of the initiation complex and halting protein synthesis. As a result, it shortens the length of peptide chains and slows down the translation process [59]. Due to its unique mechanism of action, there has been no evidence of cross-resistance with other protein synthesis inhibitors. Additionally, linezolid may inhibit the expression of virulence factors, leading to a reduction in toxin production by Gram-positive bacteria [60]. Resistance to linezolid can arise through point mutations in the 23S rDNA or through the acquisition of the *cfr*, *poxtA*, and *optrA* genes. The most prevalent resistance mechanism involves the G2576T mutation in 23S rDNA [61].

Over the past decade, the prevalence of linezolid-resistant enterococci (LRE) has surged alongside its expanded use. Reports of resistance emerging during linezolid treatment have become increasingly common. A recent systematic review and meta-analysis encompassing 84 studies published between 2004 and 2021 estimated the global prevalence of LRE at 3.3% (95% CI; 2.3–4.6%; I2 = 98.63%; *p* < 0.001). This analysis included data from 28 countries, highlighting LRE isolates found in both humans and animals. Specifically, the pooled prevalence was 1.9% (CI = 1.3–2.8%) in humans and 6.3% (CI = 3.1–12.3%) in animals [62]. In a recent investigation by Shan et al., researchers explored potential reservoirs of linezolid resistance genes in the intestines of humans and animals, as well as in meat samples and water sources. They discovered that these resistance genes were widely distributed across various environments. The horizontal transfer of these genes was identified as a key factor in the spread of linezolid resistance at the intersections of human, animal, and environmental interfaces [63]. Furthermore, data from the German Antimicrobial Resistance Surveillance revealed a rise in LRE among invasive isolates, increasing from 0.6% in 2019 to 1.2% in 2021 [53]. An analysis of *E. faecium* isolates linked to bloodstream infections in Europe from 2014 to 2018 showed a population-weighted mean proportion of 1.6% (95% CI 1.33–2.03%). This European mean is comparable to findings from various studies in other regions, including Republic of Korea, China, India, Iran, and the United States, where the prevalence remains below 2% [64].

Daptomycin, a bactericidal cyclic anionic lipopeptide that gained approval in 2003, operates through a mechanism that remains somewhat elusive. It is believed to engage with phospholipids within the cell envelope, disrupting its delicate balance. This disruption is often linked to mutations in genes that code for proteins essential for maintaining cell-envelope stability (such as *liaFSR* and *yycFG*) and those that regulate phospholipid metabolism (including *cls*, *cfa*, and *more*). Additionally, alterations in surface charge, membrane depolarization, and variations in cell wall thickness further enhance resistance to daptomycin. The LiaFSR signaling pathway is crucial in mediating daptomycin resistance in enterococci; when this pathway is nonfunctional, the bacteria exhibit increased sensitivity to daptomycin [64].

An analysis of 6949 vancomycin-resistant *E. faecium* isolates from patients with a bloodstream infection reveals a notably low rate of daptomycin resistance across Europe [64]. Interestingly, researchers have identified a phenomenon known as collateral sensitivity in vancomycin-resistant *E. faecium*, where exposure to daptomycin appears to restore susceptibility to glycopeptides. This suggests that daptomycin may effectively counteract resistance to vancomycin and teicoplanin in certain strains of VRE. However, it remains uncertain whether this collateral sensitivity is a widespread occurrence [65].

Daptomycin exposure and immunosuppression may contribute to the emergence of daptomycin resistance. However, research by El Haddad et al. revealed that even 75% of patients with daptomycin-resistant VRE had never been treated with daptomycin, indicating a likely nosocomial spread of these resistant strains within hospital settings. This theory gains traction from their findings, which show a significant genetic similarity among some of the daptomycin-resistant isolates [66]. Additionally, a study by Gargis et al. found that a substantial portion (71%, 12 out of 17) of daptomycin-resistant *E. faecium* isolates collected in the United States between 2013 and 2016 belonged to the emerging ST736 clone, exhibiting mutations in *liaFSR* and *cls* that have been linked to resistance [67]. Similarly, El Haddad et al. confirmed that all strains classified as ST736 and ST664 in their research were resistant to daptomycin, reinforcing the potential connection between these specific genetic lineages and daptomycin resistance [66].

Tigecycline, belonging to the glycylcycline class, primarily functions through mechanisms akin to that of other tetracyclines. It inhibits bacterial protein translation by reversibly binding to a specific helical region (H34) on the 30S subunit of bacterial ribosomes, effectively disrupting the elongation of the peptide chain [68].

Tigecycline-resistant *E. faecium* is on the rise, yet the exact mechanisms behind this resistance remain elusive. There is a scarcity of data concerning the molecular foundations of tigecycline resistance. Most studies indicate that resistance rates are below 1%, varying significantly across different geographical areas and species analyzed [69]. Specifically, the resistance rates for *E. faecium* strains were found to be 1.3% in Asia (95% CI 0.0–4.8), 3.9% in Europe (95% CI 0.0–14.8), and 0.3% in America (95% CI 0.0–4.0). Notably, the prevalence of these resistant strains was higher in Europe (27 out of 2048) compared to Asia (22 out of 1310) and the United States (9 out of 2776) [10]. A recent investigation by Bender et al. identified potential resistance mechanisms in a nosocomial cluster of tigecycline-resistant *E. faecium*, highlighting a deletion in the ribosomal protein gene *rpsJ* and a serine insertion that affects the transcriptional regulation of the ribosomal protection protein Tet(M) [70].

Due to the scarcity of new antimicrobial agents available for treatment, researchers are exploring innovative and alternative strategies to combat vancomycin-resistant enterococci. Some of these approaches include the following:

- antibiotic-chemoattractant conjugants
- antibody-antibiotic conjugants
- antimicrobial peptides and polymers
- bacteriophage therapy
- centyrins
- Clustered Regularly-Interspaced Short Palindromic Repeats (CRISPR) and CRISPR-Associated Genes (CRISPR/Cas)
- fecal microbiota transplantation
- nanoparticles, etc. [6]

Some exceptional challenges in antimicrobial treatment of enterococcal infections include polymicrobial infections and biofilm. A new and promising approach where enzymes are used to disrupt extracellular polymeric substance in biofilm was investigated in wound-like medium. Simultaneous use of trypsin and DNase I had a significant impact on biofilm produced by *Pseudomonas aeruginosa* and *Staphylococcus aureus* causing its degradation. Also, in the presence of these two enzymes, the minimum biofilm eradication concentrations of meropenem and amikacin were significantly decreased [71].

## 7. Infection Prevention Measures—Necessity and Efficacy

### 7.1. Transmission Dynamics of Enterococci in the Hospital Environment and Patients’ Risk Factors

A significant preliminary phase in the onset of nosocomial enterococcal infections seems to be characterized by an increased colonization density within the gastrointestinal tract. Additionally, studies have shown that the presence of VRE in the bloodstream of hospitalized individuals is often preceded by VRE establishing itself as the predominant species in their gastrointestinal flora. A systematic review and meta-analysis have revealed that patients colonized with VRE are 24 times more susceptible to developing bloodstream infections (BSIs) attributed to VRE in comparison to those who are not colonized [72,73].

The considerable VRE colonization burden is noted in patients with malignancies and is marking them as a high-risk population for colonization with this multidrug resistant pathogen. Systematic review and meta-analysis showed that 20% of patients with malignancy were colonized with VRE. Also, patients with acute leukemia were at higher risk for VRE colonization (risk ratio [RR] = 1.95; 95% CI, 1.17–3.26). The VRE colonization in patients with malignancies can be attributed to multiple factors. The first, previous use of vancomycin was associated with a 2-fold greater risk of VRE carriage and is one of risk factors for vancomycin resistance development [24,73]. In patients with hematological conditions, vancomycin is frequently administered, particularly as a component of antimicrobial therapy for those experiencing febrile neutropenia. This practice persists despite the recommendations of most existing guidelines for managing febrile neutropenia, which suggest that Gram-positive coverage should be reserved for instances of skin and soft tissue infections, suspected pneumonia caused by methicillin-resistant *S. aureus* (MRSA), or when there is no improvement within 48 h of starting Gram-negative treatment [74]. Webb and colleagues have suggested a scoring system aimed at predicting bloodstream infections caused by VRE, which takes into account various risk factors associated with the patient. These factors include VRE colonization, severe neutropenia, gastrointestinal disturbances, renal insufficiency, the use of anti-anaerobic antimicrobials, carbapenems, aminoglycosides, and cephalosporins. The scoring system allows for a maximum of nine points, and a threshold of five points significantly enhances the posterior probability of VRE bloodstream infections, increasing it nearly four-fold compared to baseline levels [75]. The documented rates of VRE colonization among patients with malignancies, along with the established association between colonization and subsequent infection, have led to investigations into the potential benefits of implementing active VRE screening in hematology and oncology departments. To date, this practice remains a subject of ongoing debate, with only a limited number of studies assessing its efficacy, yielding inconsistent findings [76,77].

The colonization of VRE within the gastrointestinal tract is notably enhanced in hospitalized individuals who have been administered antimicrobial agents, particularly cephalosporins and piperacillin–tazobactam. This phenomenon results in significant modifications to the gut microbiota. Research utilizing murine models elucidates that the administration of these antimicrobial drugs, which target Gram-negative bacteria, leads to a diminished production of REGIII by Paneth cells. The REGIII, a C-type lectin with antimicrobial activity against Gram-positive bacteria, when produced in lower quantities, creates an environment conducive to the proliferation of VRE in the gastrointestinal system [72]. Furthermore, patients treated with penicillins, aminoglycosides, and carbapenems are reported to have an elevated risk of VRE colonization [75,78].

Various additional risk factors have been identified, including renal replacement therapy (hemodialysis), advanced age (specifically over 65 years), prolonged and frequent hospitalizations, close contact with patients who are colonized or infected with VRE, admission to intensive care units, undergoing bone marrow transplants, experiencing febrile neutropenia, and the presence of urinary and intravascular catheters. Furthermore, conditions such as bedsores, altered bowel habits, cachexia, and decreased mobility have also been recognized as contributing factors to VRE colonization. This highlights the necessity for heightened vigilance regarding bedridden patients with hematological conditions. [73,79,80,81].

**Figure 2:**
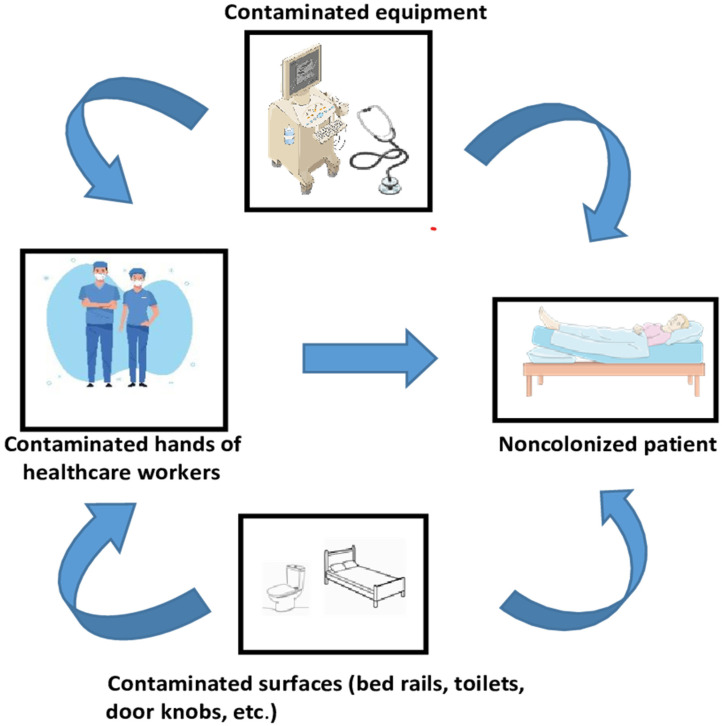
The transmission dynamics of vancomycin-resistant enterococci (VRE) within a hospital environment. The VRE is spread from colonized individuals to the surrounding area via fecal contamination. As a result, patients who are susceptible to infection may contract VRE through contact with contaminated medical equipment, surfaces, or through the inadequate hand hygiene practices of healthcare personnel. Created with Veectezy.com. This figure was also partly generated using SMART—Servier Medical Art, provided by Servier, licensed under a Creative Common Attribution 4.0 unported license https://creativecommons.org/licenses/by/4.0 (accessed on 10 September 2024).

A comprehensive understanding of the transmission dynamics of enterococci within the hospital environment is essential for effective infection control measures. The dynamics of VRE transmission in healthcare settings are illustrated in Figure 2. Patients who are not colonized can become exposed to VRE in nosocomial environments, leading to colonization in the gastrointestinal tract. Furthermore, Zhang et al. demonstrated a correlation between exposure to indoor microbiome/metabolome and the nasal/oral microbiota in pediatric populations. The presence of vancomycin resistance genes (*vanY*, *vanT*) was prevalent among these microbiota species, indicating a possible transfer of these antimicrobial resistance genes from the indoor environment to the oral cavity. Similar mechanisms could enable acquisition of the most prevalent *vanA* and *vanB* resistance genes from the hospital environment [82]. Following colonization, the pressure exerted by antibiotics has a dual impact; specifically, exposure to vancomycin promotes the survival of VRE, while the administration of various broad-spectrum antibiotics, particularly those targeting anaerobic bacteria, diminishes the protective diversity of the host’s gastrointestinal microbiome [73]. In terms of the hospital environment, VRE has been isolated from nearly every surface within patient rooms, and their widespread presence, coupled with their remarkable ability to survive on dry surfaces, contributes to elevated transmission rates of VRE in healthcare facilities. Research indicates that VRE strains obtained from the hospital environment can remain viable for over three years [83,84]. In a study conducted by Wagenvoort et al., the viability of two VRE strains isolated from a single outbreak was assessed, revealing survival durations of 1451 and 1369 days, respectively [85].

### 7.2. Resistance of Enterococci to Environmental Conditions, Disinfectants, and Sterilization Methods

Enterococci exhibit remarkable resilience in various environmental contexts. Their inherent robustness, which enables them to thrive competitively within the host gastrointestinal tract, also equips them to endure hospital environments, even in the face of rigorous sanitation protocols. This resilience facilitates their acquisition of antibiotic resistance from other microbial entities. Enterococci can withstand a temperature range of 8 to 45 °C, tolerate pH levels between 4.5 and 10.0, and endure conditions with 6.5% NaCl and 40% bile salts. These attributes clearly account for their ability to survive extreme chemical and environmental challenges that are lethal to most non-spore-forming bacteria [86]. Furthermore, their survival in hospital settings is significantly enhanced by the development of antibiotic resistance, primarily through mechanisms of horizontal gene transfer [7]. They can also endure heat exposure of up to 80 °C for 33 min and demonstrate variable resistance to sub-lethal concentrations of chemical disinfectants, including alcohol and chlorhexidine [79].

The environment plays a crucial role in the dissemination of enterococci within healthcare facilities, with environmental contamination being a significant factor in the transmission of VRE. For example, a VRE outbreak has been associated with various contaminated items, such as digital ear thermometers, rectal thermometers, and nursing bells. Enterococci can persist on hospital surfaces for extended periods, remaining viable on dry surfaces for over three years, which includes medical devices, bed rails, and doorknobs [75,76]. This persistence is critical for the transfer of these pathogens from patients or healthcare personnel to the environment and subsequently to other individuals [87]. Reports indicate that healthcare workers have transmitted VRE after interacting with contaminated surfaces, even without direct contact with colonized patients. The prolonged survival of enterococci on dry surfaces has recently been attributed to their existence in what is termed “dry-surface biofilm” (DSB), a phenomenon initially identified in MRSA. It is suggested that the majority of bacteria within DSBs exist in a dormant state with diminished metabolic activity, allowing them to evade both surface disinfection and current culture-based detection methods [88]. There is an urgent need to enhance research on the efficacy of environmental cleaning and disinfection, tailoring these studies to the emerging concept of DSBs. Most importantly, a deeper understanding of the viability of bacteria within these unconventional biofilms is essential. To enhance the effectiveness of surface monitoring and the protocols for cleaning and disinfection, it is essential to incorporate the concept of DSBs for which is believed to contain viable but non-culturable bacteria.

Research indicates that enterococci can endure prolonged periods in dry environments, with some studies reporting survival durations exceeding four months. An examination of enterococci survival on various surfaces and textiles in healthcare facilities found that the minimum survival duration was 11 days, while polyester and polyethylene exhibited longer survival times relative to other materials. Although there is a scarcity of data concerning the influence of antimicrobial resistance on bacterial longevity, recent investigations into enterococci imply that survival rates remain largely unaffected [87].

Meta-analyses have consistently demonstrated that the presence of infected or colonized individuals in a hospital room significantly elevates the risk of healthcare-associated infections caused by the same pathogen [89]. The admission of a patient free from VRE into a room previously occupied by a VRE-colonized patient has been identified as an independent risk factor for the subsequent acquisition of VRE by the newly admitted patient [90,91]. It is hypothesized that the newly admitted patient may contract VRE through contact with inadequately cleaned surfaces or via healthcare workers who may transfer VRE after touching contaminated surfaces, even without direct interaction with the discharged colonized or infected patient. A study focused on VRE colonization in intensive care units (ICUs) found that, despite rigorous double terminal cleaning protocols, 10% of the rooms still harbored residual VRE [92]. In contrast, research by Bass et al. suggested that patients placed in the same bed as a previously VRE-colonized patient did not exhibit an increased risk of VRE acquisition. The authors posited that this discrepancy might stem from variations in the effectiveness of terminal cleaning procedures, or the types of cleaning agents employed across different healthcare environments [93]. A recent investigation by Verhougstraete et al. noted a reduction in microorganism detection and concentration on surfaces from pre- to post-cleaning. However, during one sampling event in a specific room, the concentration of VRE was found to increase from below the limit of detection prior to cleaning to 0.15 CFU cm^2^ after cleaning. This finding indicates the potential for cross-contamination during the cleaning of hospital rooms and underscores the need for further research into the transmission of healthcare-associated infections (HAIs) via fomites during cleaning practices [94]. The goal of achieving pathogen eradication to levels below detection is not consistently met, despite the design, development, and marketing of terminal cleaning protocols and products aimed at this objective. The variability in cleaning effectiveness and the potential for cross-contamination remain critical concerns.

Manually applied liquid disinfectants often struggle to effectively cover all surfaces and maintain the necessary contact time within the intricate environment of hospitals. In contrast, automated room disinfection techniques typically ensure that no significant surfaces are overlooked; however, they may deliver varying and sometimes unpredictable doses of the active ingredient across different surfaces, as evidenced by recent findings regarding H_2_O_2_ nebulizers [95]. Over the past decade, the potential of automatic room disinfection devices has been explored as a means to enhance surface disinfection. Current recommendations for the use of these automated systems emphasize the importance of conducting microbiological verification tests within the specific clinical setting to validate their effectiveness. When implementing a new automated system or disinfecting a newly designed room, it is advisable to perform microbiological culture tests (where permitted) or environmental swab tests both before and after disinfection to assess efficacy. It is crucial to recognize that recent clinical isolates may exhibit different behaviors compared to laboratory strains, which are typically utilized during the approval of disinfection methods. For instance, a comparison of the reduction rates of ten distinct clinical isolates of VRE with *E. hirae* ATCC 10541—a standard test strain for airborne automatic room disinfection—revealed that some clinical VRE isolates demonstrate greater stability against UV-C radiation than the commonly used test organism [95]. Furthermore, a meta-analysis indicated that hydrogen peroxide vapor (HPV) was linked to statistically significant decreases in the incidence of VRE infections or acquisitions when compared to ultraviolet C (UV-C) and pulsed-xenon ultraviolet (PX–UV) systems. This may be indicative of the persistence of VRE in clinical settings and the capacity of HPV to effectively reach all surfaces, whereas UV light may be hindered by shadowing effects, among other factors [96].

Current cleaning techniques frequently fall short in effectively managing biofilm contamination, particularly in relation to dry surface biofilms within healthcare settings. Consequently, there is a pressing need to explore innovative technologies aimed at eliminating biofilm contamination on hospital surfaces. Some researchers propose cold atmospheric pressure plasma (CAPP) as a viable decontamination method, demonstrating efficacy against bacteria in dry surface biofilms through the action of reactive oxygen species (ROS) and reactive nitrogen species (RNS). However, further evaluation against established disinfection standards is still necessary [97].

### 7.3. Are Contact Precautions for Patients with Vancomycin-Resistant Enterococci Still Necessary?

Preventive measures designed to mitigate the emergence and transmission of multidrug-resistant microorganisms within healthcare environments encompass the following key strategies: implementation of hand hygiene protocols, adherence to contact precautions (CPs), thorough environmental cleaning, communication within and between hospitals when patient is transferred, promotion of antimicrobial stewardship, detection of colonized individuals of patients, and education of healthcare personnel on adherence to recommended interventions [98].

Recent evaluations have called into question the rationale behind CP aimed at preventing the transmission and colonization of hospitalized patients with VRE. While these measures were once deemed reasonable and necessary, advancements in our understanding of infection prevention strategies over the past several decades have prompted some researchers to reassess the necessity of CP for patients colonized with VRE. Despite the prevalent implementation of CP, there is limited evidence supporting its effectiveness in preventing MRSA or VRE infections in endemic environments. Concurrently, the application of CP has significant implications for patient care and safety [99]. A thorough systematic review of the literature has identified various adverse outcomes associated with CP, including a reduction in the time healthcare workers spend with patients, delays in admission and discharge processes, heightened levels of anxiety and depression among patients, and diminished patient satisfaction with care [100]. Furthermore, patients subjected to CP are six times more likely to encounter adverse events during hospitalization, such as falls and pressure ulcers. In comparison to non-isolated patients, those under MRSA CPs experience a 30% increase in length of hospital stay and a 43% rise in healthcare costs [101].

A systematic literature review and meta-analysis encompassing 14 studies found that the cessation of contact precautions (CPs) for patients with endemic MRSA and VRE did not correlate with an increase in infection rates. In the United States, an increasing number of hospitals are re-evaluating their CP protocols for this patient demographic. Instead, they are redirecting their efforts towards horizontal infection control measures, which include hand hygiene, bare-below-the-elbows attire, chlorhexidine bathing, care bundles, and environmental sanitation. This systematic review indicates that these hospitals have not experienced immediate rises in infection rates despite the absence of gowns and gloves when entering patients’ rooms [11,12].

In the research conducted by Martin and colleagues, CPs for MRSA and VRE were terminated at 12 hospitals within the UPMC health system, while three other hospitals maintained these precautions. The researchers gathered and evaluated the rates of MRSA and VRE HAIs over a 12-month period, both prior to and following the implementation of these changes. The findings revealed that the aggregated HAI rates from the hospitals that discontinued contact precautions were 0.14 and 0.15 MRSA HAIs per 1000 patient days (*p* = 0.74), and 0.05 for both VRE HAIs per 1000 patient days (*p* = 0.96), as well as 0.04 for MRSA laboratory-identified events per 100 admissions (*p* = 0.57). These results indicated no statistically significant differences in rates between the hospitals that ceased or continued the precautions. It is noteworthy that the hospitals that achieved successful outcomes had low baseline rates of MRSA and VRE HAIs, along with high compliance in hand hygiene practices. Therefore, hospitals contemplating the removal of contact precautions for MRSA and VRE must first ensure that their rates of these infections are low, and that hand hygiene compliance is robust before making such a decision [102]. Additionally, a study by Eichel et al. found that the cessation of contact precautions did not lead to an increase in VRE transmission, nor was there a rise in the mean incidence density of VRE bacteremia, as basic hygiene measures were concurrently enforced [103]. To our knowledge, the study with the longest duration of discontinuing contact precautions for VRE positive patients was the study by Lemieux et al. The study was conducted in four large academic hospitals, the duration period was 3.5 years, and there was no impact on VRE healthcare-associated infection rate [104].

The reasons behind the lack of change in infection rates following the cessation of CP remain ambiguous. One potential explanation is that CPs may not be significantly effective in preventing endemic infections caused by MRSA and VRE, leading to the conclusion that their discontinuation does not influence infection rates. Alternatively, it is possible that other effective measures, such as enhanced hand hygiene practices, are adopted in place of CPs once they are no longer implemented. It is important to note that these findings do not extend to epidemic situations, and there is a lack of research assessing the consequences of halting CPs for resistant gram-negative pathogens or *Clostridioides difficile*. The cessation of CPs, as currently employed for MRSA and VRE, can be achieved; however, this requires a robust horizontal infection prevention strategy that emphasizes high compliance with hand hygiene practices [11].

Current evidence suggests that it is prudent to cease the implementation of contact precautions (CPs) for MRSA and VRE in healthcare settings characterized by low endemic rates and high compliance with hand hygiene practices. Concurrently, it is essential to advocate for horizontal prevention strategies, which encompass hand hygiene, antimicrobial stewardship, and effective environmental cleaning techniques aimed at reducing colonization rates. Nevertheless, contact precautions for MRSA and VRE should remain in place to mitigate transmission during uncontrolled outbreaks, particularly in patients exhibiting open wounds, uncontained secretions, or episodes of incontinent diarrhea [99]. Recent studies indicate a growing preference for a patient-centered approach to contact precautions, as opposed to overly broad policies that may jeopardize patient safety.

### 7.4. Value of Active Screening for VRE in Detection of Colonized Patients and Documenting the Clearance

Active screening or surveillance for VRE has been utilized for several decades to facilitate the early identification of colonized or infected individuals. The primary objective of this approach is to implement CPs promptly and mitigate the transmission of these pathogens to other patients. Research indicates that identifying patients colonized with multidrug-resistant organisms, coupled with the enforcement of CP protocols, can significantly lower the rates of colonization and infection associated with these pathogens. A systematic review conducted by the ECDC aimed at assessing the efficacy of control measures to reduce the incidence of colonization or infection with carbapenem-resistant Enterobacterales (CRE) found that the most effective strategies included CPs, early screening for CRE-colonized patients, and cohort nursing for those patients [105]. Outbreaks caused by various multidrug-resistant pathogens, including VRE, have been effectively managed using this strategy in conjunction with other infection control practices. However, more recent investigations have raised concerns regarding the efficacy of active surveillance through screening, particularly in environments where the multidrug-resistant pathogen is endemic [106].

There is a lack of agreement regarding the most effective protocols for screening, CP, and surveillance of VRE, as evidenced by the differing infection control strategies observed both within and across various countries. To see the reports published in the last five years (from January 2019 until the present), a search was made of the literature in PubMed using the terms “vancomycin resistant” in combination with the terms “screening”, and “active surveillance” in the title of the report in the last five years. Reports regarding active surveillance of VRE in the last five years are summarized in Table 3.

A review of the literature available on PubMed reveals that 14 studies concerning active screening for VRE have been published in the past five years. The majority of these studies (11 out of 14) indicate that VRE screening is both beneficial and cost-effective in hospitals with a high prevalence of VRE, particularly during outbreak situations. Conversely, such screening may not be warranted in facilities with low prevalence rates [76,107,108,110,111,113,114,115]. Notably, research conducted by Cho et al. found a significant rise in the incidence of healthcare-associated VRE bacteremia following a change in VRE screening policy, with this increase predominantly affecting high-risk patient groups, including those in hematology–oncology and intensive care units [46]. In settings characterized by low endemicity, prioritizing the screening of high-risk patients—such as those in hematological care, individuals in intensive care, patients who have been in close contact with VRE-colonized or infected individuals (patients in the same room with a VRE colonized/infected patient), and patients taken care of by the same healthcare workers—emerges as the most effective strategy for identifying VRE transmission [112,116]. Additionally, active screening may be employed temporarily to assess VRE prevalence and the ratio of intestinal colonization to infection [68].

The VRE colonization can be detected by stool, rectal, and perirectal swabs, all of them representing sensitive methods. Various factors can compromise the sensitivity of culture methods and chromogenic agar. These include a MIC of vancomycin for VRE isolates, a decreased concentration of VRE in the fecal specimen, and high concentration of vancomycin in the culture media [119]. The *vanA* and *vanB* genes occur in various bacterial species beyond *Enterococcus* spp. (*Streptococcus mitis*, *Streptococcus bovis*, *Eubacterium lenta*, *Ruminococcus* spp., *Lactococcus* spp., *Leuconostoc* spp., certain species of *Clostridium* spp.). This influences specificity and positive predictive value (PPV) of nucleic acid amplification tests (NAATs) [120]. Significant levels of *vanB* gene in gastrointestinal tract of both healthy and ill individuals can be observed. Elevated rates of *vanB* carriage of nonenterococcal origin were detected among community adults (63%), children (27%), and patients undergoing hemodialysis in a study by Graham et al. (27%). *vanB* gene present in *Enterococcus* spp. and those found in other bacterial species cannot be distinguished by the PCR detection methods [121].

Stool, rectal, and perirectal swabs represent effective techniques for identifying VRE colonization. The sensitivity of culture methods and chromogenic agar can be diminished due to several factors, including a low vancomycin minimum inhibitory concentration (MIC) of the VRE isolates being tested, a reduced fecal density of VRE in the sample, and elevated concentrations of vancomycin in the culture media [116]. Additionally, the low specificity and positive predictive value (PPV) of nucleic acid amplification tests (NAATs) can be attributed to the presence of *vanA* and *vanB* genes in various bacterial species beyond *Enterococcus* spp. These include *Streptococcus mitis*, *S. bovis*, *Eubacterium lenta*, *Ruminococcus* spp., *Lactococcus* spp., *Leuconostoc* spp., and certain *Clostridium* species [117].

The sensitivity of a single rectal swab is relatively low, with reported values ranging from 42.5% to 79%. This sensitivity improves when multiple swabs are collected. Several factors contribute to this variability, including fluctuations in the fecal excretion of VRE, differing concentrations of VRE in feces, and the time interval required for VRE to become detectable in samples following transmission. The duration of VRE colonization varies across studies, with median durations reported between 26.5 days and over six months [122]. The number of rectal cultures necessary to classify a known carrier or contact patient as VRE-negative remains ambiguous [113]. According to Dutch national guidelines, it is recommended to collect 3 to 5 rectal cultures on separate days, with the final culture ideally taken at least seven days after the last exposure [123]. However, this approach may impose significant burdens on laboratory costs and hospital capacity, particularly for patients awaiting results from three screening cultures. Therefore, it may be prudent to reassess this protocol in healthcare facilities with lower complexity of care, especially when the last exposure occurred more than seven days prior [113].

## 8. Conclusions

A notable increase in the global prevalence of vancomycin resistance, alongside the emergence of resistance to linezolid, daptomycin, and tigecycline, underscores the critical need for effective infection control strategies targeting this group of enterococci. The notable resilience of enterococci to various environmental conditions highlights the necessity for investigations into innovative technologies capable of effectively targeting the biofilm produced by enterococci on hospital surfaces. A critical approach to traditional infection control strategies is becoming more accepted worldwide taking into account epidemiological situations in the given healthcare setting as well as the specific characteristics of a patient. For certain high-risk patient populations, traditional infection control strategies, including CP and screening, should not be omitted. Additionally, further investigation into the resistance mechanisms of available antimicrobial agents is essential, as is research into their potential association with specific successful clones through WGS genotyping, to preemptively mitigate their spread before it escalates.

## Figures and Tables

**Table 1:** Vancomycin resistance genes and their associated phenotypes.

Vancomycin Resistance Gene	Level of Resistance to Vancomycin and Teicoplanin	Part of the World in Which Genotype Is Detected
*vanA*	high-level of resistance to both vancomycin and teicoplanin	worldwide [27,29]
*vanB*	high-variable resistance to vancomycin susceptibility to teicoplanin	worldwide [27,29]
*vanC*	low-level resistance to vancomycin susceptibility to teicoplanin	worldwide [27,29]
*vanD*	low- to high-level resistance to vancomycin and teicoplanin	France, The Netherlands, Canada, Australia, North America, Japan, Sweden, Brazil [27,29,30,31]
*vanE*	low-level resistance to vancomycin, susceptibility to teicoplanin	Norway, North America, Australia [27,29]
*vanG*	low-level resistance to vancomycin susceptibility to teicoplanin	Australia, Canada [27,29]
*vanL*	low-level resistance to vancomycin susceptibility to teicoplanin	Canada [27,29]
*vanM*	high-level resistance to vancomycin and teicoplanin	China, Singapore [27,29]
*vanN*	low-level resistance to vancomycin susceptibility to teicoplanin	France [27,29]

**Table 2:** The most important virulence genes, their mechanisms of action, and significance in pathogenesis of enterococcal infection.

Virulence Determinants	Encoding Genes and Their Location	Mechanism of Action	Significance in Pathogenesis of Infection
Aggregation substance	*asa1* (plasmid)	surface protein adhesion; enables the conjugative transfer of sex pheromone gene-containing plasmids through clumping of one *Enterococcus* to another [43]	increases bacterial adherence to renal tubular cells and heart endocardial cells; in animal models, increases the valvular vegetation mass [43]
Gelatinase	*gelE* (chromosomal)	extracellular zinc endopeptidase that hydrolyzes collagen, gelatin, and small peptides [41,43]	exacerbates endocarditis in an animal model [43]
Cytolysin	*cylA* (plasmid or chromosome)	cellular toxin; consists of two components—lysin (L) and activator (A); *cylA* is necessary for the expression of component A; lyses a range of prokaryotic and eukaryotic cells [43]	significantly worsens the severity of endocarditis and endophthalmitis in animal models; contributes to the severity of enterococcal disease in humans [43]
Enterococcal surface protein	*esp* (chromosome)	the central repeat region extends the N-terminal globular domain through the cell wall to the surface [41,43]	increases virulence, colonization, and persistence in the urinary tract; plays role in adherence to abiotic surfaces and biofilm formation [41,43]
Hyaluronidase	*hyl* (chromosome)	homology to hyaluronidase previously described in *Streptococcus pyogenes*, *Staphylococcus aureus*, and *S. pneumoniae* [43]	acts on hyaluronic acid and increases bacterial invasion [43]
Collagen-binding protein	*acm* (unknown)	adhesin of collagen from *E. faecium*; cell-surface adhesins shown to recognize and adhere to various components of the extracellular matrix [44]	endocarditis model results provide experimental evidence that *acm* contributes to *E. faecium* pathogenesis [44]

**Table 3:** Reports regarding active screening of vancomycin-resistant enterococci from 2019 until the present.

Authors (Year)	Country	Type of Study/Number of Patients or Samples Included	Patients Who Were Screened	Conclusion Regarding the Need for VRE Screening
Hertz et al. (2022) [107]	Denmark	Single center study/99 consecutive rectal swabs	Patients admitted to the Department of Neuroanaesthesiology	Yes for a hospital with a high VRE prevalence, but may not be justified in the hospitals with a low prevalence
Mac et al. (2019) [108]	Canada	Microsimulation model with base-case analysis/over 1000 admissions	Simulated general medicine ward	The VRE screening can be considered a cost-effective infection prevention and control intervention in this simulation study. The intervention’s cost-effectiveness varied depending on VRE prevalence and isolation effectiveness.
Chhatwal et al. (2020) [76]	Germany	Prospective study/2052 samples were collected and 213 were positive for VRE	Adults with hematological malignancies	A screening protocol was implemented for a duration of one year to assess the VRE prevalence and to evaluate intestinal colonization/infection ratio. Nevertheless, universal screening upon admission and/or weekly may not be essential, particularly while VRE infections continue to be rare.
Stordeur et al. (2024) [109]	France	Retrospective study/256 patients	10 healthcare centers	A predictive score was created that shows high performance for the assessment of XDR clearance; no conclusions regarding VRE screening
Telli Dizman et al. (2024) [110]	Turkey	Retrospective observational study/total of 12,524 patients	Adults admitted to the intensive care unit and internal medicine ward	Screening policies ought to be tailored to the specific epidemiological context of VRE, alongside considerations of healthcare workers’ capacity and financial implications. Regular rectal screening for VRE may be suspended if there is a robust system in place for monitoring potential outbreaks.
Pfister et al. (2022) [111]	Canada	Intervention time-series Poisson regression	Acute care facilities	There was no observed increase in the incidence of HA VRE–BSI in locations or units that ceased screening for VRE, irrespective of the risk group of the patients
Büchler et al. (2022) [112]	Switzerland	Retrospective study/1096 patients	Patients after exposure to VRE-infected or colonized patients in tertiary care hospital	In a low-endemic environment, prioritizing the screening of high-risk patients who have been exposed, as well as those receiving care from the same medical team, seems to be the most effective strategy for identifying transmissions of VRE.
Weterings et al. (2021) [113]	The Netherlands	Retrospective description of interventions	All patients admitted to the hospital	A control strategy that integrated targeted screening and isolation, along with the application of general precautions and environmental cleaning, effectively managed the outbreak.
Hansen et al. (2024) [114]	Denmark	Retrospective e cohort study/436 patients	Screening of contact patients during and after an outbreak	This research examined the consequences of discontinuing VRE screening and isolation protocols at a Danish university hospital. Although there was an increase in the number of patients presenting with a first-time clinical VRE isolate, the findings did not indicate any necessity for reinstating screening and isolation measures.
Heininger et al. (2020) [115]	Germany	Prospective study/3265 samples from 2572 high-risk patients	Admission screening of patients at risk for multidrug-resistant organisms	The authors conclude that, given the low prevalence and minimal risk of infection associated with endogenous VRE, there is currently no pressing need to implement routine VRE screening in our hospital.
Cho et al. (2022) [46]	Republic of Korea	Retrospective time-series analysis/364 HA-VRE bacteremia cases in 360 patients	Screening practices only for high-risk units including the hemato-oncology ward, pediatric ward, and medical ICUs	The incidence of HA–VRE bacteremia increased significantly after the VRE screening policy change, and this increase was mainly driven by high-risk patient populations such as patients in the hemato-oncology department and ICUs.
Eichel et al. (2020) [116]	Germany	Retrospective study/43 patients	Patients with bacteraemia and routine VRE screening in high-risk patients at admission, and weekly in ICUs	The evaluation of high-risk patients during the admission screening process may enhance the understanding of the scale of an outbreak within an endemic context.
Kram et al. (2021) [117]	United States	Retrospective cohort study/855 patients with an enterococcal BSI	patients with an enterococcal BSI	Facilities that have established VRE screening protocols for infection control may incorporate VRE rectal swabs into antimicrobial stewardship initiatives within the ICU population. In clinical practice, a greater positive predictive value would be necessary to justify the implementation of a universal screening approach for all patients in the ICU.
Wammes et al. (2023) [118]	The Netherlands	Retrospective observational study/135 patients with culture positive for VRE	Patients with culture positive for VRE	The goal of research was to ascertain the optimal quantity of screening cultures required to enhance the sensitivity for detecting VRE transmission, as well as to establish the duration from the time of exposure to the point of detectable colonization. The study concluded that both the frequency of swabs collected and the timing of these swabs following exposure are critical factors.

Abbreviations: VRE, vancomycin-resistant enterococci; HA, healthcare-associated; BSI, bloodstream infection; ICU, intensive care unit.

## Data Availability

No new data were created or analyzed in this study. Data sharing are not applicable to this article.
